# Correction: A long-persistent phosphorescent chemosensor for the detection of TNP based on CaTiO_3_:Pr^3+^@SiO_2_ photoluminescence materials

**DOI:** 10.1039/c8ra90040j

**Published:** 2018-05-21

**Authors:** Fangfang Li, Fengyi Wang, Xuan Hu, Baozhan Zheng, Juan Du, Dan Xiao

**Affiliations:** College of Chemistry, Sichuan University 29 Wangjiang Road Chengdu 610064 China dujuanchem@scu.edu.cn; Key Laboratory of Green Chemistry and Technology, Ministry of Education, College of Chemistry, Sichuan University Chengdu Sichuan 610064 China

## Abstract

Correction for ‘A long-persistent phosphorescent chemosensor for the detection of TNP based on CaTiO_3_:Pr^3+^@SiO_2_ photoluminescence materials’ by Fangfang Li *et al.*, *RSC Adv.*, 2018, **8**, 16603–16610.

In Fig. 2(C) of the published paper the colours of the lines were switched. A correct version of the figure is shown below:

**Fig. 2:**
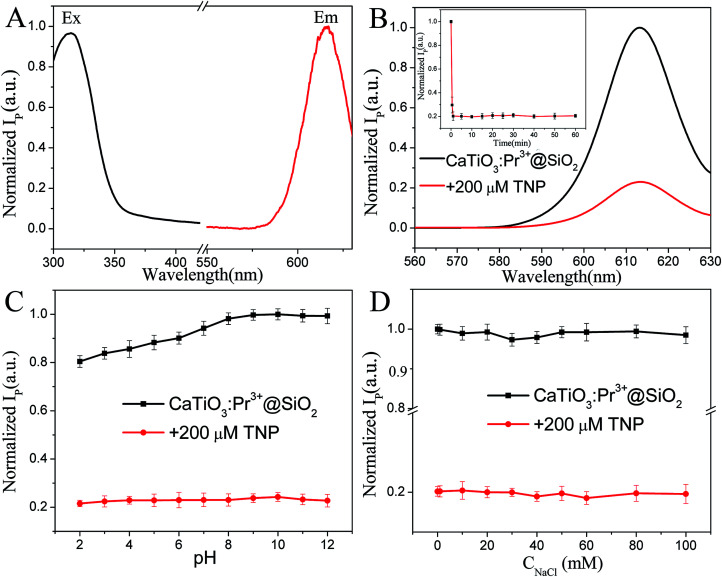
(A) Phosphorescence excitation (Ex) and emission spectra (Em) of CaTiO_3_:Pr^3+^@SiO_2_ (30 μg mL^−1^) in PBS solution (10 mM, pH = 8.0), (*λ*_Ex_ = 315 nm, *λ*_Em_ = 614 nm). (B) Phosphorescence spectra of 30 μg mL^−1^ CaTiO_3_:Pr^3+^@SiO_2_ with (red curve) and without (black curve) 200 μM TNP. Inset: temporal change in the phosphorescence intensity of CaTiO_3_:Pr^3+^@SiO_2_ after the addition of TNP. Effect of (C) pH and (D) salt concentration on the phosphorescence intensity of CaTiO_3_:Pr^3+^@SiO_2_ (30 μg mL^−1^) in the absence (black line) and presence (red line) of 200 μM TNP. *λ*_Ex_ = 315 nm (error bars, SD, *n* = 3).

The Royal Society of Chemistry apologises for these errors and any consequent inconvenience to authors and readers.

## Supplementary Material

